# Abstracts From the First Annual JADPRO Live Transforming Oncology Practice Meeting

**Published:** 2014-03-01

## Abstract

Renaissance Vinoy, St.Petersburg, Florida January 24-26, 2014

## JL01. The Role of the Multidisciplinary Tumor Board in the Management of Patient with Gastrointestinal Malignancy

Steve Malangone, MSN, NP-C, Nurse Practitioner, Hematology/Oncology, University of Arizona Cancer Center

The optimal evaluation and management of the patient with gastrointestinal (GI) malignancy is multimodal, involving coordination between multiple specialties, including radiology, pathology, medical oncology, surgical oncology, radiation oncology, interventional radiology, gastroenterology, and nursing. Furthermore, accurate staging and pathologic evaluation is essential in the selection of appropriate management, and involves input from pathology, diagnostic imaging, interventional endoscopy, and medical oncology. A major challenge in delivering coordinated care to patients with GI malignancies is in communication and coordination between various specialists. Differences in practice location, delays in the availability of completed diagnostic reports and progress notes, and a lack of dynamic two-way communication in the traditional clinic setting threaten the efficient delivery of the best-practice combined-modality cancer therapy to the patient. Management guidelines from the National Comprehensive Cancer Network (NCCN) recommend multidisciplinary evaluation in the management of esophageal, esophagogastric junction, gastric, hepatobiliary, pancreatic, colon, and rectal cancers. An effective approach to enhance communication is the multidisciplinary cancer conference (MCC). In the MCC, members of each discipline meet in real time in a designated location and via video/teleconferencing. The patient is presented with joint review of actual medical data including history, radiographic and endoscopic images, and pathology. Each member of the team provides input in the assessment of the case, and available treatment modalities are discussed with a focus on the development of an individualized consensus approach for the patient in accordance with current best practice. Outcomes related to MCCs include improved patient outcomes (both in overall survival and in decreased time between diagnosis and treatment), planning, survival, patient satisfaction, and clinician satisfaction in cooperation/communication. The author of this presentation has had an opportunity to both attend the conference and present patients, and believes that the advanced practice nurse (APN) can experience professional growth and provide added value to the MCC. The focus of this poster will be on providing an overview of MCCs with an emphasis on the role of the APN. A patient case from the University of Arizona Cancer Center in Tucson will be included to illustrate ways in which MCCs enhance patient outcomes. The role of each member will be briefly included, with examples related to patient outcomes. 

## JL02. Virtual Reality as an Adjunct Method to Alleviate Pain and Anxiety in Patients Undergoing Bone Marrow Procedures

Kelly Young, DNP, RN, AOCN, Duke University Medical Center; Susan M. Schneider, PhD, RN, AOCN, FAAN, Duke University School of Nursing; Pandora Lassiter, PharmD, Duke University Medical Center; and Louis Diehl, MD, Duke University Department of Medicine, Durham, North Carolina

*Purpose: * The purpose of this randomized controlled study is to determine the clinical feasibility of using virtual reality at the bedside with patients who are undergoing a bone marrow biopsy. Outcome measures included pre and post pain and anxiety assessment and cost analysis. Methods: A prospective, randomized control trial design was used to test three hypotheses. The first was that virtual reality (VR) immersion can be successfully applied to patients undergoing bone marrow biopsy and aspirates. The second was that patients receiving VR immersion experience less pain and anxiety than without VR. The third hypothesis was that VR immersion is a cost-effective adjunct in clinical care. Results: VR was successful at the bedside during bone marrow biopsies. While not statistically significant, the VR intervention did show a trend toward better outcomes in both pain control and anxiety. VR also has the potential to result in a fiscal advantage over standard care. VR was well received and provided distraction for the majority of patients. Conclusions: VR immersion is a user-friendly intervention that subjectively provides distraction for patients who are undergoing bone marrow biopsy and aspirates. It is a relatively inexpensive intervention in today’s technological environment. Virtual reality as an intervention deserves additional research in clinical settings.

## JL03. Adding to Your Tool Box: Holistic Modalities

Marilyn Haas, PhD, APN-C, and Sheri Denslow, PhD, MPH

Significance: Holistic medicine, as defined by the American Holistic Medical Association, "is the art and science of healing that addresses care of the whole person—body, mind, and spirit." The practice of holistic medicine integrates conventional/traditional medicine and complementary therapies to promote optimal well-being, and prevent and treat disease by addressing underlying factors. Advanced practitioners can offer adjunctive, effective, and evidence-based techniques to treat symptoms associated with cancer and related treatments. A growing body of scientific evidence supports integrative modalities in oncology by numerous health-care providers. Many modalities can be learned and offered to oncology patients to increase their quality of life while undergoing therapies. Purpose: The purpose of this research study was to investigate the effects on the parasympathetic nervous system with four holistic modalities offered to oncology patients by certified holistic nurses. The secondary aims were to identify which holistic modalities would be chosen by oncology patients during hospitalization or radiation treatments, examine the changes in physiologic vital sign measurements (blood pressure, pulse, respiration), and correlate patients’ self-reported evaluation of anxiety, nausea, and pain with the modalities received. Interventions: Holistic consults were offered to oncology patients in the hospital/radiation therapy department by physicians and nurses. Patients could select any or all of the four modalities. Vital signs and self-evaluation of three potential symptoms (anxiety, nausea, pain) were assessed prior to beginning the modality and at completion. Evaluation: After receiving IRB approval, 442 patients participated in this study. Available modalities (healing touch, guided imagery, aroma therapy, massage) were explained, and verbal consent was obtained. Sixty-seven percent chose healing touch first, followed by guided imagery (23%) and hand/foot massage (21%), with aroma therapy being the least selected modality (8%). After receiving any of these interventions, statistically significant drops in physiologic vital sign measurements were observed with each modality (t-test *p* < .01) except aroma therapy (*p* = .49 on diastolic blood pressure). Changes in self-reported improvement in anxiety, nausea, and pain were statistically significant (*p* < .01) except aroma therapy (*p* = .08 for nausea). Discussion: Holistic interventions play an increasingly important role in controlling symptoms associated with cancer treatments/hospitalizations. Teaching and incorporating three integrative modalities into clinical practice by advanced practitioners can improve symptomatology, and promote self-care. Advanced practitioners can learn these modalities through qualified CEU courses, as most curriculums do not include integrative modalities. 

## JL04. Adjuvant Treatment With Toremifene in Hormone Receptor–Positive Breast Cancer: A Systematic Review of the Literature

Carolyn Lavender, MSN, AOCNP, ARNP, ProStrakan Inc, Bridgewater, New Jersey; Marcelle Kaplan, RN, MS, AOCN, CBCN, Adelphi University College of Nursing and Public Health, Garden City, New York; and Deborah Braccia, RN, MPA, PhD, ProStrakan Inc, Bridgewater, New Jersey

Background: Endocrine therapy is an integral part of the backbone for hormone receptor–positive breast cancer treatment. Toremifene and tamoxifen are approved for hormone receptor–positive metastatic breast cancer. Tamoxifen is also approved for use in adjuvant breast cancer; however, tamoxifen may not be appropriate for all patients. The purpose of this presentation is to review and assess available data on the efficacy and safety of toremifene in the adjuvant treatment setting. Methods: Systematic review of the literature. Results: Five prospective trials, one retrospective trial, and a meta-analysis have been published regarding the use of toremifene vs. tamoxifen in the adjuvant setting. A combined analysis of 1,035 women enrolled in two different trials showed similar disease-free survival (DFS), overall survival (OS), and recurrence rates (RR) after a mean 5.5 years of follow-up. A separate study showed similar time to recurrence, risk of recurrence, and OS rates with 3 years of therapy with tamoxifen or toremifene. Another study in patients with 5 years of therapy demonstrated equivalent OS with a median follow-up of 59 months. A recent phase III prospective study in postmenopausal women reported noninferiority of adjuvant treatment with toremifene compared with tamoxifen. Both treatments were safe and well tolerated, with no significant differences in the frequency or severity of adverse events, in each of these adjuvant trials. A retrospective study in premenopausal women examined records from 452 patients and showed similar OS with improved DFS with adjuvant toremifene compared to tamoxifen. Finally, a recent meta-analysis including 3,747 patients reported similar OS, DFS, and safety in patients given adjuvant tamoxifen or toremifene. Conclusion: A series of published studies indicate toremifene is as effective and safe as tamoxifen in the adjuvant setting. 

## JL05. Efficacy and Safety of the Granisetron Transdermal System (GTS) in Elderly Patients Receiving Multiday Chemotherapy Regimens

Paula J. Anastasia, Cedars-Sinai Medical Center, Los Angeles; Vanna M. Dest, Smilow Cancer Hospital at Yale New Haven, New Haven, Connecticut; Christine Brown, ProStrakan Inc; Doris Wong, ProStrakan Inc; and Deborah Braccia, ProStrakan Inc, Bridgewater, New Jersey

Background: Chemotherapy-induced nausea and vomiting (CINV) is a common side effect of multiday chemotherapy that can lead to poor adherence, decreased quality of life, and compromised functional status. Commonly used routes of administration of antiemetics, such as oral and intravenous, are accompanied by a number of challenges. Elderly patients compound challenges with oral agents due to the added risk of nonadherence owing to dementia or impaired vision. This analysis examined the impact of age on the efficacy and safety of a > antiemetic, the Granisetron Transdermal System (GTS), in elderly cancer patients receiving multiday chemotherapy. A published, randomized phase III trial (N = 641) demonstrated the noninferiority of GTS vs. oral granisetron in complete CINV control in adult patients receiving multiday chemotherapy and served as the basis of its FDA approval in the prevention of CINV in patients receiving moderately and/or highly emetogenic chemotherapy regimens of up to 5 consecutive days duration. Methods: This post-hoc analysis divided patients from the previously reported phase III study into three age categories: < 50 years of age, 50–64 years of age, or ≥ 65 years of age (defined as elderly). The primary endpoint was the percentage of patients achieving complete control of CINV (no vomiting and/or retching, no more than mild nausea, and no use of rescue medication) from the first administration until 24 hours after final administration of chemotherapy. Additional endpoints were the use of rescue medication; the percentage of patients achieving complete response (no vomiting and/or retching, no rescue medication); patients’ global satisfaction with antiemetic therapy (assessed using a 10-cm visual analog scale (VAS) at the time of patch removal); and safety. Results: A total of 621 patients were included, 142 of whom were elderly. There were no statistically significant differences in baseline characteristics between treatment groups. No significant differences were found in complete control rates, complete response, use of rescue medication, or patients’ global satisfaction with antiemetic treatment between the GTS and oral granisetron treatment groups regardless of age. GTS was well tolerated with mild side effects; gastrointestinal disorders were the most common study drug-related treatment-emergent adverse events in each age subgroup of the GTS-treated population. Conclusion: This post-hoc analysis showed the efficacy and safety of GTS were maintained in all age subgroups, suggesting that GTS is an appropriate antiemetic agent for a wide range of ages including the elderly.

## JL06. Hereditary Hemochromatosis: What the Nurse Practitioner Needs to Know

Kathy Leonard, RN, NP-C, AOCNP®, NYU Langone Medical Center, New York, New York

Background: Hereditary hemochromatosis is a disorder in which excessive iron absorption occurs, leading to an increase in iron deposits in major organs of the body and resulting in end organ damage. This is the most common genetic disorder in individuals of Northern European ancestry, and the history of this disease can be traced back to the time of the Vikings and Celtics. Although it was known for many years that this was a hereditary disease presenting with the classic triad of diabetes, bronze pigmentation of the skin, and cirrhosis of the liver, it was not until 1996 that the HFE gene was identified. Hereditary hemochromatosis is an autosomal recessive disorder where a mutation occurs at on chromosome 6 of the short arm of HFE resulting in two changes: the substitution of tyrosine for cysteina at position 282 (C282Y) and the substitution of aspartic acid for histidine at position 63 (H63D) of HFE protein. Hemochromatosis is easily treated if identified early, before it manifests itself as a disease. The treatment is therapeutic phlebotomy. Discussion: Individuals who have this mutation are asymptomatic until their ferritin levels are markedly elevated, at which point penetrance of the disease may present in affected organs. The disease may manifest itself as diabetes, arrhythmia, liver disease, arthritis, and sexual dysfunction. It is most important that primary care nurse practitioners have a familiarity with the hereditary hemochromatosis and how to screen, diagnose, and treat patients with the disease.

## JL07. Intravenous Thiotepa for Meningeal Relapse of Breast Cancer: A Case Study

Kathryn Clarke, RN, MSN, FNP-C

Treatment of meningeal carcinomatosis among breast cancer patients remains a clinical challenge, with a dismal outlook for long-term survival. Intrathecal thiotepa administration has been described in the literature; however, there are practical challenges of administration into the intrathecal space, and there is no significant difference in drug exposure as measured by AUC between intravenous and intrathecal routes. One female with meningeal relapse of HER2-positive breast cancer hospitalized and felt to be preterminal was treated with IV thiotepa in 2010. Her neurologic status returned to baseline within 3 weeks, and she was felt to be in a complete radiologic and clinical remission, which continues to present day. This compelling case suggests a role for IV thiotepa in the treatment of breast cancer patients with meningeal carcinomatosis, and further study is warranted. 

## JL08. Merkel Cell Carcinoma: Is It on Your Differential?

Paige M. Goforth, MMS, PA

Background: Merkel cell carcinoma (MCC) is an aggressive neuroendocrine cutaneous malignancy with an atypical presentation, associated with the merkel cell polyomavirus (MCPyV) in 80% to 90% of cases. The vowel mnemonic, AEIOU is representative of components of both risk factors and tumor characteristics: asymptomatic, expanding rapidly, immune compromised, older than age 50, and ultraviolet light exposure. The incidence has quadrupled over the past 20 years, particularly in immunocompromised individuals. Metastatic MCC has a median survival rate of 9.6 months. Early detection is critical, as the 5-year survival rate increases to nearly 80% with prompt treatment. The major implication for oncology advanced practitioners is to recognize risk factors in those currently under their care, especially those who are immunocompromised from chemotherapy and/or radiation. Risk factors include age older than 50, immunocompromised, and ultraviolet light exposure/fair skin. If the patient is older than 50, they now have the two risk factors that are seen with the highest percentage in patients presenting with a primary merkel cell tumor. Skin rashes or lesions are often associated with oncologic treatments and the recognition of risk factors and tumor characteristics of MCC may lead to earlier detection. There is an estimated 15.7-fold increased risk of MCC following chronic lymphocytic leukemia (CLL) diagnosis and 17-fold increased risk of CLL following MCC diagnosis. MCC should be in the differential for a reaction following treatment for CLL. Pathology is required to determine if a lesion is an aggressive malignancy vs. a reaction to chemotherapy. Biopsy is critical in patients who exhibit AEIOU risk factors presenting with a suspicious lesion reported as painless with red, dome shaped, and rapidly growing. Patients with a confirmed diagnosis of merkel cell carcinoma should be referred to a multidisciplinary team for clinical decision making and coordination of care. Treatment options for merkel cell carcinoma include wide surgical excision, adjuvant radiation, immunotherapy, and chemotherapy for metastatic disease.

## JL09. Personalized Medicine for Breast Cancer

Beverly Rhodes, RN, MSN, ANP, The University of Texas MD Anderson Cancer Center, Houston, Texas

Breast cancer is a biologically complex disease and remains one of the leading causes of cancer-related deaths in the world. Recent advances in genomic profiling of breast cancer tumors show promise in improving the outcomes of breast cancer patients. Testing the molecular composition of breast cancer tumors is a practice that began years ago with the testing and use of the estrogen receptor status of breast cancer tumors to guide the use of endocrine therapy. More recently, the practice of genetic profiling of the tumor has provided clinicians with a much greater opportunity to provide molecular-based personalized medicine. It has facilitated improvement in the prognostication of distant disease recurrence and the prediction of the benefit of adjuvant chemotherapy. This has allowed many patients to avoid unnecessary toxicities associated with chemotherapy. Studies have shown that gene assays have influenced clinicians to change treatment recommendations in 25% of patients. Molecular profiling can also provide information about agents that are most likely to have the greatest clinical benefit and identify those that have a potential lack of benefit. The identification of aberrant genes in breast cancer tumors can help guide clinicians and pharmaceutical companies in the development of targeted therapeutic agents. The possibility of monitoring a patient’s disease status, response to a therapy or early detection of relapse represents other areas where personalized medicine may prove beneficial. Circulating tumor cells (CTCs) can be used to monitor treatment effect in metastatic breast cancer and provide additional prognostic information for patients on active therapy. CTCs have a comprehensive molecular characterization that could possibly serve as a "liquid biopsy," thus enabling targeted therapy. Research is ongoing to identify other reliable tumor markers. The molecular profiling of breast cancer and its implications are promising. Many studies of personalized medicine in breast cancer are ongoing, and many more are needed to bring this exciting approach to the clinical forefront. 

## JL10. Subcutaneous Administration of Bortezomib: A Pilot Survey of Oncology Nurses

Jasmine R. Martin, DNP, MSN, APRN, Director, Scientific Alliances and Research, Millennium: The Takeda Oncology Company; Nancy L. Beegle, BSN, Yanyan Zhu, PhD, and Joan M. Agretelis, PhD

Background: The subcutaneous (SC) route of administration for bortezomib was approved in the United States in January 2012 in addition to the intravenous (IV) route. Although there is limited guidance in the US Prescribing Information regarding how to administer SC bortezomib, more broadly there is a lack of clear direction in the literature on administering SC chemotherapy. Studies in non-oncology fields suggest that injection site reactions, pain, and bruising can be reduced by using short needles injected at 45 degrees into skin folds, practicing the air bubble rather than purge technique, and slower injections. Inconsistent or poor administration techniques may increase injection site reactions and could contribute to patients stopping effective treatment. It is important to identify how oncology nurses are administering SC bortezomib. This observational survey of oncology nurses practicing in community oncology clinics aimed to identify the techniques used and explore nurses’ opinions about SC bortezomib administration. Methods: A 44-question electronic survey on SC bortezomib administration was developed, with all questions based on current literature regarding appropriate techniques for administering SC injections. Questions were also included regarding convenience of SC administration, nurses’ opinions on patients’ preferences for administration site, and correlation between facility layout and administration site Results: A total of 43 oncology nurses from 17 clinics responded to the survey. Advanced Practice Nurses (APRNs) shared the responsibility with oncologists for ordering SC bortezomib according to 53% of respondents. Nurses reported using the abdomen (98%), thigh (19%), and arm (53%) for SC bortezomib. Nurses used 25 gauge 5/8 inch (42%) and 27–30 gauge ≤ 1/2 inch (56%) needles, with both 45 degree (61%, 42%) and 90 degree (39%, 58%) angles of insertion used with these respective needle sizes (p=0.21). The air purge technique was used by 49% of nurses, with 51% using an air bubble technique. Nurses took 3–5 (49%), 5–10 (35%), 10–30 (9%), and > 30 seconds (7%) to administer each milliliter of SC bortezomib injection. Conclusions: These findings indicate the heterogeneity of techniques used for SC bortezomib administration. APRNs may consider evaluating SC injection techniques used in practice settings, even where guidelines are in place, in order to promote consistent caring practice and professional evidence-based collaboration.

## JL11. Don’t Fall for It! Preventing Falls in Patients with Cancer

Glenda Kaminski, PhD, RN, CNS, AOCN, Clinical Nurse Specialist, Oncology, Lakeland Regional Medical Center, Lakeland, Florida

The Oncology Nursing Society’s 2009–2013 research agenda highlights the need for greater attention to impaired physical functioning in inpatients with cancer because of the significant physical, psychological, and economic consequences. Fall prevention is an important nursing quality indicator, but fall prevention programs for hospitalized patients have had limited success. The purpose of this oncology unit-based initiative was to identify factors that place patients with cancer at greater risk for falling and to explore whether the Schmid scale is able to identify those patients at highest risk to fall or sustain an injury should they fall. A retrospective review of all falls that occurred between February 1 and September 30, 2013 provided insight into factors that might place a patient with cancer into a high risk for falls category compared to other inpatients. The two most common environmental factors that the patients who fell had in common were being tethered by IV or oxygen tubing and/or using a bedside commode. Patient factors included age, gender, disease process, treatment, and length of stay. The Schmid did not appropriately identify oncology patients at risk for falling. A total of 14 of 37 patients had low risk scores prior to their falls. Additionally, staff nurses need to have a better understanding of how to utilize the scale in a more consistent manner. Advanced practice nurses need to review data related to falls in patients hospitalized with cancer to devise and test evidence-based fall prevention strategies. They also need to educate staff nurses to improve consistency in use of screening tools.

## JL12. Management of Lenalidomide-Associated Cytopenias in Myelodysplastic Syndromes: Practical Take-Aways From Clinical Trials

Sandra E. Kurtin, RN, MS, AOCN®, ANP-C, University of Arizona Cancer Center, Tucson; Jean A. Ridgeway, MSN, APN, NP-C, AOCN®, University of Chicago Medical Center, Chicago; Sara Tinsley, ARNP, MS, AOCN®, H. Lee Moffitt Cancer Center and Research Institute, Tampa, on behalf of the MDS Foundation International Nurse Leadership Board

Lenalidomide (LEN) is an oral immunomodulatory medication approved for the treatment of patients with transfusion-dependent anemia due to low- or intermediate-1‒risk myelodysplastic syndromes (MDS) with del(5q). In clinical trials of LEN in MDS, neutropenia and thrombocytopenia were common; severe myelosuppression was generally managed with dose modifications rather than discontinuations, similar to our clinical experience. If treatment is discontinued too early, patients may not receive enough medication to decrease transfusion needs. Additionally, we have observed patients with sustained, mild-to-moderate, asymptomatic cytopenias that do not need intervention. This is unlike other malignancies in which neutropenia and thrombocytopenia are significant events requiring treatment discontinuation. Therefore, it is important to anticipate and manage LEN-associated cytopenias to extend treatment and optimize outcomes. In this report, MDS-003 and MDS-004 clinical trial data are applied to real-world patient care to illustrate expected cytopenias and outline strategies for practical management of LEN-related cytopenias in MDS patients. Rates, time to onset/recovery, and LEN dose modifications due to neutropenia or thrombocytopenia were examined. These data and expert experience were used to prepare a practical guide for management of cytopenias in LEN-treated MDS patients relevant to advanced practitioners in oncology. Management of cytopenias in LEN-treated MDS patients is dependent on baseline blood counts, bone marrow function, treatment cycle, severity, and symptoms. In the MDS-003/004 trials, rates of grade 3/4 neutropenia and thrombocytopenia were higher in early treatment cycles but decreased thereafter. Severe cytopenias were generally transient and managed primarily with dose reductions and interruptions. A complete blood count (CBC) with differential and platelet count is suggested weekly in the first 8 weeks of treatment when cytopenias are expected and may require temporary dose interruption. LEN should be interrupted and dose reduced for platelet counts < 50,000/µL in cycle 1, but in later cycles, platelet counts as low as 30,000/µL are tolerated in the absence of a need for platelet transfusions or bleeding abnormalities. Unlike in other hematologic malignancies, sustained moderate but asymptomatic cytopenias may persist over months or years with continued transfusion independence and no need for dose modification in the absence of symptoms or decreases in quality of life (Kurtin, 2012). Familiarity with expected cytopenias, planning for effective management, and setting expectations for the patient and family will support the advanced practitioner in management of cytopenias and promote continued therapy in the MDS patient achieving transfusion independence in response to LEN.

## JL13. Practical Guide to Management of Lenalidomide-Related Rash in Patients With MDS

Sara Tinsley, ARNP, MS, AOCN, H. Lee Moffitt Cancer Center and Research Institute, Tampa; Sandra E. Kurtin, RN, MS, AOCN, ANP-C, University of Arizona Cancer Center, Tucson; Jean A. Ridgeway, MSN, APN, NP-C, AOCN, University of Chicago Medical Center, Chicago, on behalf of the MDS Foundation International Nurse Leadership Board

Lenalidomide (LEN) is an oral immunomodulatory medication approved for patients with lower-risk, transfusion-dependent myelodysplastic syndromes (MDS) with del(5q) with or without additional cytogenetic abnormalities. The goal of LEN treatment is to reduce or eliminate red blood cell transfusion dependence. A recent analysis of the Celgene Global Drug Safety database showed that nonserious rash was the leading cause of permanent early discontinuation of LEN in MDS in the postmarketing setting. The majority of discontinuations due to rash occurred in < 2 cycles (8 weeks) of treatment. In clinical trials, rash led to no or low rates of discontinuation. LEN rash is generally self-limiting, resolving within 2–3 weeks without adjustment of LEN treatment. This suggests differences in real-world management of rash vs clinical trials. It may take ≥ 3 cycles of LEN treatment to achieve transfusion benefit. Therefore, early recognition and proper management of rash by advanced practitioners in oncology may reduce morbidity and extend treatment to optimize outcomes of LEN-treated patients with MDS. This practical guide to management of LEN-related rash in patients with MDS encompasses identification of physiology, signs, and symptoms of LEN-related rash, grading of rash, recommended rash management guidelines, and patient communication tips. Most LEN-related rash is mild-to-moderate and may present as patchy, raised, macular skin lesions, sometimes with localized urticaria, which may be associated with pruritus. Cutaneous reactions may be associated with immunomodulatory properties of LEN and usually require no intervention. Patients with mild-to-moderate rash (grade 1/2; covering ≤ 30% of body surface area [BSA]) may be treated with topical corticosteroids and/or oral antihistamines until it is grade ≤ 1 or resolves. For grade 3 rash (covering > 30% of BSA), interrupting LEN and treating with oral antihistamines or short courses of oral corticosteroids is recommended. LEN treatment can generally be restarted after interruption without rash reoccurrence. LEN must be permanently discontinued for life-threatening rash (grade 4), angioedema, exfoliative or bullous rash, or if Stevens-Johnson syndrome or toxic epidermal necrolysis is suspected. Patients can be further educated using rash photos and explanations of how rash is treated in advance, and encouraged to promptly report signs of skin problems. Advanced practitioners can effectively manage LEN-related rash by being aware of symptoms, applying appropriate levels of intervention, and involving patients in self-reporting early signs of rash through upfront educational initiatives.

## JL14. Practical Guide to Bone Marrow Sampling for Suspected Myelodysplastic Syndromes

Jean A. Ridgeway, MSN, APN, NP-C, AOCN, University of Chicago Medical Center, Chicago; Sara Tinsley, ARNP, MS, AOCN, H. Lee Moffitt Cancer Center and Research Institute, Tampa; and Sandra E. Kurtin, RN, MS, AOCN, ANP-C, University of Arizona Cancer Center, Tucson, on behalf of the MDS Foundation International Nurse Leadership Board

Over 10,000 individuals are diagnosed with myelodysplastic syndromes (MDS) annually in the United States. Bone marrow (BM) examination is essential for diagnosis, classification, and risk-stratification of MDS. The World Health Organization classification is based on BM blast percentage and type and degree of dysplasia. Risk stratification using the Revised International Prognostic Scoring System requires blasts percentage, depth of cytopenias, and characterization of cytogenetic abnormalities. Proficiency in this procedure is critical to obtain high-quality BM specimens to facilitate accurate diagnoses and minimize patient discomfort and risk. The BM examination requires a BM aspirate (BMA) to evaluate cell morphology and cellular elements, including blasts, and core BM trephine biopsy (BMTB) to describe cellularity, topography, stromal elements, and the proportion and maturation of hematopoietic cells. Combined examination of the BMA and BMTB allows the most thorough morphological assessment. Assessing dysplasia can be difficult, thus specimen quality is critical. Quality depends on the instruments used as well as operator proficiency. Generally, two adjacent sites are sampled to obtain the BMA and BMTB to avoid crush artifact and poor sampling. The posterior iliac crest is the preferred site for this procedure. Patients are positioned prone or in the right/left lateral decubitus position. Following sterile site preparation, the periosteum is infiltrated with 5 to 10 mL of 1% to 2% lidocaine to minimize pain. BMA samples should be evaluated for the presence of spicules to ensure proper BM sampling. If a BMA cannot be obtained due to fibrosis or cellular packing, touch preparation of the BMTB can yield valuable cytologic information. An adequate BMTB specimen must be ≥ 1.5 cm in length to allow evaluation of ≥ 10 partially preserved intertrabecular areas. Following the procedure, firm pressure is applied to the site for 5 minutes for adequate hemostasis, followed by placement of a pressure dressing. Patients at risk of bleeding (thrombocytopenia, aspirin use, and anticoagulation) should be evaluated, and if necessary treated, prior to the procedure to reduce bleeding risk. The patient should be instructed not to bathe, swim, or soak in a Jacuzzi for at least 48 hours after the biopsy. Complications are unusual but patients should be given an emergency contact number in case of bleeding, pain, fever, erythema, or swelling at the biopsy site. The pressure dressing may be removed after 24 hours and acetaminophen may be taken for pain. 

